# Agreement between Gonioscopic Examination and Swept Source Fourier Domain Anterior Segment Optical Coherence Tomography Imaging

**DOI:** 10.1155/2016/1727039

**Published:** 2016-11-20

**Authors:** Mohammed Rigi, Nicholas P. Bell, David A. Lee, Laura A. Baker, Alice Z. Chuang, Donna Nguyen, Vandana R. Minnal, Robert M. Feldman, Lauren S. Blieden

**Affiliations:** ^1^Robert Cizik Eye Clinic, 6400 Fannin St., Suite 1800, Houston, TX 77030, USA; ^2^Ruiz Department of Ophthalmology and Visual Science, McGovern Medical School at The University of Texas Health Science Center at Houston (UTHealth), 6431 Fannin St., MSB 7.024, Houston, TX 77030, USA

## Abstract

*Purpose*. To evaluate interobserver, intervisit, and interinstrument agreements for gonioscopy and Fourier domain anterior segment optical coherence tomography (FD ASOCT) for classifying open and narrow angle eyes.* Methods*. Eighty-six eyes with open or narrow anterior chamber angles were included. The superior angle was classified open or narrow by 2 of 5 glaucoma specialists using gonioscopy and imaged by FD ASOCT in the dark. The superior angle of each FD ASOCT image was graded as open or narrow by 2 masked readers. The same procedures were repeated within 6 months. Kappas for interobserver and intervisit agreements for each instrument and interinstrument agreements were calculated.* Results*. The mean age was 50.9 (±18.4) years. Interobserver agreements were moderate to good for both gonioscopy (0.57 and 0.69) and FD ASOCT (0.58 and 0.75). Intervisit agreements were moderate to excellent for both gonioscopy (0.53 to 0.86) and FD ASOCT (0.57 and 0.85). Interinstrument agreements were fair to good (0.34 to 0.63), with FD ASOCT classifying more angles as narrow than gonioscopy.* Conclusions*. Both gonioscopy and FD ASOCT examiners were internally consistent with similar interobserver and intervisit agreements for angle classification. Agreement between instruments was fair to good, with FD ASOCT classifying more angles as narrow than gonioscopy.

## 1. Introduction

A crucial part of any evaluation for the primary angle closure (PAC) spectrum of diseases is an examination of anterior chamber angle (ACA) anatomy. Gonioscopy is the current clinical gold standard for evaluating ACA anatomy, allowing assessment of the angle over 360 degrees as well as identification of other angle characteristics, such as peripheral anterior synechiae (PAS) and pigmentation level. However, gonioscopy is difficult to perform and subjective, limiting its use in clinical research.

A variety of imaging technologies have been developed to evaluate ACA anatomy in a more quantitative manner. Anterior segment optical coherence tomography (ASOCT) is widely used because it is quick and noncontact and provides reproducible images of the angle, and image acquisition can be performed by a technician with minimal training [1–5]. However, most ASOCT instruments measure only horizontal and vertical meridians, with up to 80% of superior angles not adequately visible [6]. This may limit the clinical application of ASOCT, because, on gonioscopy, the narrowest angle, which is considered as the most important clinically, is the superior angle [7].

While there have been several published studies comparing angle classification using ASOCT to clinical gonioscopy [6–12], the agreements are widely variable, in large part due to the image quality and difficulty imaging the superior angle. The CASIA SS-1000 ASOCT (Tomey Corporation, Nagoya, Japan) uses Fourier domain (FD) swept source technology to provide high-resolution images (10 *μ*m axially and 30 *μ*m transversally for 2D images) for both horizontal and vertical meridians in 1.2 seconds. From our prior studies, we have found that this device provides high-quality images of the superior angle that can be reliably analyzed using customized software [3–5, 13].

The purpose of this study was to evaluate the interobserver, intraobserver (intervisit), and interinstrument agreements for both gonioscopy and the CASIA SS-1000 FD ASOCT for evaluation of the superior angle in both open and narrow angle eyes.

## 2. Participants and Methods

This prospective cohort study was conducted at the Robert Cizik Eye Clinic of the Ruiz Department of Ophthalmology and Visual Science at the McGovern Medical School at The University of Texas Health Science Center at Houston (UTHealth). Institutional Review Board approval was obtained from The University of Texas Committee for the Protection of Human Subjects before study commencement. All research adhered to the tenets of the Declaration of Helsinki and was HIPAA compliant. Informed consent was obtained from all study participants before initiation of study data collection and procedures.

### 2.1. Participants

Consecutive adult participants (at least 18 years old) who visited the Robert Cizik Eye Clinic were recruited. Participants were excluded if they used any medication that may have affected angle anatomy within a month before imaging (e.g., pilocarpine or atropine). Eyes were excluded if there was a history of penetrating trauma, any intraocular procedures within 90 days before imaging, anticipated intraocular procedures before completion of the second study visit, or any anterior segment abnormalities affecting visualization of the angle (e.g., significant corneal opacity). When both eyes of the participant were eligible, one eye was randomly selected by a coin flip (heads: right eye, tails: left eye).

**Figure 1:**
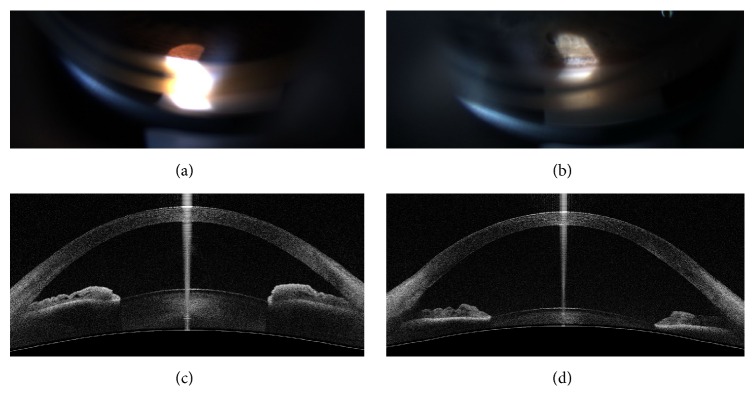
Comparison of a narrow angle and wide-open angle. (a) Gonioscopy image of a narrow angle. (b) Gonioscopy image of a wide-open angle. (c) 2D anterior segment optical coherence tomography (ASOCT) image (vertical) from the CASIA SS-1000 of a narrow angle. (d) 2D ASOCT image (vertical) of a wide-open angle. (a, c) Images from the same eye; (b, d) Images from the same eye.

After obtaining informed consent, demographics, ocular history, and present ocular medications were recorded, and slit lamp examination was performed to screen for eligibility. Cataracts were graded without dilation to avoid angle closure in narrow angle participants. Eligible participants underwent a gonioscopy examination and FD ASOCT imaging (first visit). At the second visit, within 6 months of the first one, gonioscopy with the same examiners and FD ASOCT imaging were repeated (Figure 1).

### 2.2. Gonioscopy

The study eye was examined by gonioscopy on 2 separate visits by 2 of 5 glaucoma specialists (NPB, LSB, DAL, VRM, and RMF) on the same day. Gonioscopy was performed using a Posner 4-mirror lens at high magnification (10x), with the eye in the primary position of gaze under the lowest possible ambient lighting conditions by turning off all ambient light sources and closing the door of the exam room. Gonioscopic examination was first performed without indentation, with care taken to minimize light from the slit lamp beam from entering the pupil. The superior quadrant was graded for iris insertion using the Spaeth grading system (grading the deepest visible ACA structure, A: anterior to Schwalbe's line; B: between Schwalbe's line and scleral spur; C: scleral spur; D: ciliary body; and E: beyond 0.1 mm of ciliary body) [14, 15]. Gonioscopy was then performed with indentation for grading presence or absence of PAS at 12 o'clock, 3 o'clock, 6 o'clock, and 9 o'clock. During the second visit, the gonioscopic examinations were performed by the* same 2 examiners* from the initial visit in the* same order* (1st or 2nd examiner). Examiners were masked to the grading of the other examiners. Spaeth grading from the gonioscopy exam without indentation was used for the agreement study.

### 2.3. FD ASOCT Instrument and Acquisition of FD ASOCT Images

Details of the CASIA SS-1000 FD ASOCT and procedures for image acquisition have been previously described [4]. Eyes were scanned in 2D mode in the dark (lighting in the room was measured at 0 lux) using the angle analysis scan type with the autoalignment function.

### 2.4. FD ASOCT Image Reading

All raw study images were exported from the FD ASOCT after data collection was complete and imported to customized software, Anterior Chamber Angle and Interpretation (ACAI, Houston, TX), as described in previous publications [3–5]. Two experienced FD ASOCT readers (AZC, LAB) identified scleral spur landmarks (SSLs) on* superior* angles (12 o'clock) and graded iris insertion using Spaeth definitions (A, B, C, D, or E). Magnification and contrast adjustment of the image were allowed to clarify the position of the SSLs and Schwalbe's line. When Schwalbe's line was not visible, the measurement rings that mark 250, 500, and 750 *μ*m from the SSL were turned on. An angle was graded as “A” if the iris insertion was 500 *μ*m anterior to the SSL, as the length of the trabecular meshwork is approximately 500 *μ*m [16, 17]. Both readers examined all study images independently and were masked to the gonioscopy evaluation and FD ASOCT results from the other reader.

### 2.5. Sample Size and Power Calculation

In an effort to have sufficient participants representing the whole spectrum of angle grades, the study recruited 15% A, 15% B, 20% C, 30% D, and 20% E angles, as determined using the grading from the first examiner at the first visit. A minimum of 70 participants was required for kappa = 0.4 agreement, with a precision of 0.15 on each side of the agreement. With an estimated 80% of participants expected to return for the second visit, 88 participants needed to be recruited.

### 2.6. Data Analysis

Demographics were summarized by mean and standard deviation (SD) for continuous variables or by frequency (%) for discrete variables. Angles were classified using grading from both examinations (gonioscopy and FD ASOCT) as “narrow” if graded A or B and as “open” if graded C, D, or E.

Kappa statistics were calculated for the angle classification to evaluate* interobserver agreement* between gonioscopic examiners and agreement between FD ASOCT readers at each visit. The agreement between gonioscopic examiners at each visit was calculated by pooling all 1st examiners versus pooling all 2nd examiners, as the sample size was too small to evaluate the interobserver agreement for* each* of the 10 different pairs of gonioscopy examiners.* Intraobserver (intervisit) agreement* was calculated between visits for each pair of gonioscopy examiners (5 of them) and combining all 5 examiners for each ASOCT reader;* interinstrument agreement* was determined for each examiner-reader pair (with examiner pooled 1st or 2nd) at each visit.

All statistical analyses were performed using SAS for Windows version 9.4 (SAS, Inc., Cary, NC). The kappa criteria were <0.2 poor; 0.21 to 0.40 fair; 0.41 to 0.60 moderate; 0.61 to 0.80 good; and >0.80 excellent [18].

## 3. Results

A total of 88 eyes of 88 participants were recruited. Two eyes were excluded, one participant withdrew consent, and images could not be obtained from the other participant, leaving a total of 86 eyes enrolled in the study. Seventy-two participants (84%) returned in a mean of 1.2 months (SD = 1.1 months, range 1 day to 4 months) for the second visit, which was more than the anticipated 80% returning for the second visit. Demographics and baseline ocular characteristics are summarized in Table 1. One participant's FD ASOCT image (visit 1) was not analyzed due to poor image quality; another participant was imaged by FD ASOCT at visit 2 but did not undergo gonioscopy. All sequential gonioscopy was performed with at least 3 minutes to 480 minutes between exams. Any sequential gonioscopy and then ASOCT imaging was performed with at least 5 minutes between exam and testing.

### 3.1. Gonioscopy Agreement

Table 2 shows “pooled”* interobserver* agreements for gonioscopy among examiners. The agreement was* good* (kappa = 0.66 and 0.69 at visits 1 and 2, resp.). Pairs of gonioscopy examiners agreed on angle classification in 85% and 89% of eyes for visits 1 and 2, respectively.* Intraobserver* (intervisit) agreements ranged from* moderate* (kappa = 0.53) to* excellent* (kappa = 0.86) for individual examiners and good (kappa = 0.74) for all examiners combined. Ninety percent (79 eyes) were classified into the same group (open or narrow) on both visits (Table 3).

### 3.2. ASOCT Agreement

The FD ASOCT readers had* moderate* to* good interobserver* agreement (kappa = 0.73 and 0.58 at visits 1 and 2, resp.) (Table 4). Both readers agreed on angle classification in 74 (87%) eyes at the first visit and 58 of 72 (81%) eyes in the second visit. FD ASOCT reader 1 had* moderate intraobserver* agreement (kappa = 0.57) while FD ASOCT reader 2 had* excellent* intraobserver agreement (kappa = 0.83). Of the 14 angle disagreements between visits by reader 1, 4 eyes were graded as “C” in visit 1 and “B” in visit 2, while 6 eyes were graded as “C” in visit 1 and “B” in visit 2.

### 3.3. Gonioscopy and FD ASOCT Agreement

The* interinstrument* agreements between the “pooled” gonioscopy examiners and FD ASOCT readers ranged from* moderate* to* good* (kappa ranged from 0.42 to 0.63, Table 5). The percentage of agreement for angle classification between each examiners-reader pair ranged from 71% to 82%. In general, more eyes were classified as “narrow” by the FD ASOCT readers than by the gonioscopy examiners.

## 4. Discussion

Gonioscopy is the current clinical gold standard for evaluation of the ACA, but it is difficult to perform and a subjective method for examining the angle. Angle grading depends on examiner interpretation, which limits the potential for repeatable quantitative measurements using gonioscopy alone. Our study found that, 10% of the time, the same examiner disagreed with their original gonioscopic classification for the same patient seen at the second visit* (intervisit)*. Having a reproducible, objective, and quantitative method for measuring the angle could advance clinical research and ultimately improve patient care.

ASOCT has been used to evaluate angle anatomy quantitatively [19] and holds the potential to be useful for longitudinal angle evaluations. Prior studies have shown moderate or worse agreements between ASOCT and gonioscopy [6–8, 11, 12, 20]. However, a big limitation of those studies has been the ability to image the superior angle, which is the narrowest quadrant and typically used clinically (by gonioscopy) to determine whether a patient has open or narrow angles. Often, the methodology in the published literature on ASOCT and gonioscopy does not indicate which angle specifically was used for the gonioscopic classification. When indicated, most published studies compared superior quadrant gonioscopy with nasal/temporal meridian ASOCT images, which cannot be assumed to be equivalent, especially in eyes with PAC [8, 10, 11]. A few studies have compared gonioscopy to superior/inferior angles with visible scleral spur but have excluded 20–95% of imaged eyes [6, 7, 9, 12, 20].

In our study, we compared the classification agreements of the superior angles using both gonioscopy and FD ASOCT and found that interobserver agreements were* moderate* to* good* for both gonioscopy and FD ASOCT, and intraobserver (intervisit) agreements were* moderate* to* excellent* for both instruments. The agreements between instruments (gonioscopy versus FD ASOCT) for both parameters were* fair* to* good*.

### 4.1. Gonioscopy: Interobserver and Intraobserver Agreement

The interobserver agreement for gonioscopy was* good* (kappa = 0.66 and 0.69). These results are similar to results published by Hu et al. on angle classification as open or closed by 3 different gonioscopy examiners (kappa = 0.65 for superior quadrant). Hu et al. also reported a near perfect agreement (Kendall W = 0.83) using clinical assessment for* angle closure risk* [6], which takes into account not only angle classification but also other angle features assessed by gonioscopy (e.g., level of pigmentation, presence of PAS). This indicates that there is likely a complex multifactorial relationship between* angle classification* and* angle closure risk.* This relationship deserves further study.

Combined intraobserver (intervisit) agreement was* good* (kappa = 0.74) in our study. With the technical limitations of gonioscopy, we thought this might be an interesting parameter to evaluate the consistency of the individual examiner on different visits with the same patient, which was higher than the agreement reported by Campbell et al. (kappa = 0.29), the only reference we found for a study evaluating intraobserver agreement for gonioscopy [10]. Given the coordination it took to evaluate intraobserver agreement, we can understand why there is a paucity of existing literature. The less-than-perfect agreement not only points out the potential limitations of gonioscopic interpretation but also may represent the dynamic anatomic variation that may occur in the individual patient.

### 4.2. FD ASOCT: Interobserver and Intraobserver Agreement

The interobserver agreements between FD ASOCT readers were* moderate* (kappa = 0.58) to* good* (kappa = 0.73). Our definition of open or narrow angles on FD ASOCT is based on the level of iris apposition relative to the scleral spur landmark [4], which approximates the location of the scleral spur. A previous study by Quek et al. evaluated the interobserver agreement on identifying angle structures, including scleral spur, using the Cirrus (Carl Zeiss Meditec, Dublin, CA) and iVue OCTs (Optovue Corporation, Fremont, CA). In that study, the agreement for identifying scleral spur on the superior angle was poor (kappa = 0.04) for Cirrus and moderate (kappa = 0.44) for iVue [12]. In another study, Tay et al. reported that the interobserver kappa was 0.51 using the temporal/nasal angle images obtained from the Visante ASOCT instrument (Carl Zeiss Meditec, Dublin, CA) [8]. Compared to these prior studies using Cirrus, Visante, and iVue OCTs, our agreements (kappa = 0.58 and 0.73) are better.

*Intraobserver* (intervisit) kappas were 0.57 and 0.83 for readers 1 and 2, respectively, in our study, which was better than previously published results (kappa = 0.47) using the spectral domain Topcon OCT (Topcon Europe Medical BV, Netherlands) in the anterior segment mode (840 nm wavelength) [10].

Quek et al. used a set of 20 images from the Cirrus and 20 from iVue OCTs and evaluated angle structure visibility (i.e., scleral spurs, trabecular meshwork) on the same images twice, resulting in good to excellent agreement for the Cirrus OCT and excellent agreement for the iVue OCT on the visibility of each structure [12]. This study evaluated only reader variability while our study evaluated a combination of reader, device, and imaging session variabilities, because we looked at images of the same patient taken on separate visits. Given this methodology, one would expect our study to have less* intraobserver* (intervisit) agreement.

We believe that our FD ASOCT agreements are better than the previously published literature because the CASIA SS-1000 FD ASOCT produces images with less artifact that could be enhanced using customized ACAI software. Furthermore, we took a closer look at intraobserver agreement by reader 1 and found 10 of 14 disagreed angles were graded as “C” in one of the visits and “B” in the other visit. We suspect that this is due to the difficulty in evaluating C angles on the images, which may be represented by only 1-2 pixels on the screen. The inherent variability between the image, device, and observer judgment is unknown and may represent more than 1-2 pixels.

### 4.3. Interinstrument Agreement: Gonioscopy versus FD ASOCT

In our study, the majority of interinstrument agreements between FD ASOCT and gonioscopy were* moderate* (kappa between 0.41 and 0.60). Previous studies have published inconsistent results when evaluating interinstrument agreement of gonioscopy and anterior segment imaging devices. The agreements ranged from poor (kappa < 0.20) [6, 8, 11] to fair (kappa = 0.21 to 0.40) [6–8, 12] to moderate (kappa = 0.41 to 0.60) [7, 9, 12, 20]. We believe that the reason why prior studies have shown variable agreements between ASOCT and gonioscopy is because they were unable to identify angle landmarks sufficiently for classification, especially in the superior angle. Many studies reported only being able to identify a portion of landmarks in images necessary to classify the angle, resulting in those images (80 superior angles) being eliminated from the analysis [6]. Our FD ASCOT readers could visualize and identify SSL in 99% of superior angles, regardless of angle configuration. Possible explanations for the observed differences in SSL visualization between ASOCT technologies include (1) the higher scan speeds and resolution of FD ASOCT, allowing visualization of the peripheral angle with less artifact, or (2) use of the custom-designed ACAI software to enhance the visibility of the angle structures by manipulating image contrast. It is important to note that our FD ASOCT readers also found the SSL more challenging to identify in the superior and inferior angles compared to the nasal/temporal angles, consistent with other reports using ASOCT [6, 7].

### 4.4. Limitations

There are a few limitations to consider. Despite standardized dark background conditions, slit lamp illumination required for gonioscopy may induce pupillary constriction, resulting in the apparent opening of the angle or dynamic changes in iris configuration [7, 20]. Similarly, accidental indentation resulting from gonioscopy artificially opens the drainage angle [7]. In such cases, appositional closure may go undetected by gonioscopy. In fact, our FD ASOCT readers classified more angles as narrow than did our gonioscopy examiners. This is consistent with the reported literature [7, 21]. Although gonioscopy has traditionally been the gold standard for grading ACAs, given the intraobserver (intervisit) agreements found in our study and those published by Campbell et al. [10], it is not clear that it is a more reliable representation of the true angle status as open or narrow when compared to ASOCT, which may be the result of examiner technique or dynamic anatomic variation in the individual.

Another explanation for differences found between gonioscopy and FD ASOCT is related to the actual locations of measurement. Gonioscopy does not grade at a single axis, but as an area, which may be a clock hour or a quadrant, while with the FD ASOCT, measurement is taken in a single meridian. It might be appropriate in a future study to compare a quadrantric measurement, such as a quadrant of trabecular-iris circumference volume (TICV [5]), to gonioscopy rather than a single plane measurement. Unfortunately, that was beyond the scope of the current study as FD ASOCT measurements were taken with 2D images, which cannot be used for TICV.

ACA grading based on FD ASOCT imaging is not yet entirely automated; hence, readers' subjectivity might have influenced the results, especially with adjudicating the location of the scleral spur landmark. However, we previously reported good reproducibility in identifying SSL location on FD ASOCT imaging at the superior angle by 2 observers; that is, mean differences were 20 *μ*m and 7 *μ*m for the *x*-axis and *y*-axis, respectively [4]. In addition, eyelid manipulation is necessary for superior angle imaging and may have led to inadvertent changes in angle configuration. A standardized imaging protocol is in place to reduce the image variability. Finally, for practical reasons, we did not use fixed pairs of gonioscopy examiners for all participants; however, each participant was examined by the same pair of examiners in the same order for both visits. Our sample size was too small to analyze agreements between the 10 potential pairs of examiners.

In conclusion, our study demonstrates the expected agreements between ASOCT imaging and gonioscopy classification of the superior angle in open and narrow angle eyes. Previously, published studies have included limited data from the superior angle. For angle classification, ASOCT of the superior angle performs similarly to gonioscopy; however, other parameters that determine angle closure risk need further evaluation.

## Figures and Tables

**Table 1:** Demographics and baseline ocular characteristics.

Variable	Statistics (*N* = 86)
Age, years (SD)	50.9 (18.4)
Sex, *n* male (%)	27 (31%)
Race, *n* (%)	
White	34 (40%)
Black	19 (22%)
Hispanic	20 (23%)
Asian	13 (15%)
Study eye, *n* right (%)	46 (53%)
Iris color, *n* (%)	
Brown	68 (79%)
Blue	16 (19%)
Green/hazel	2 (2%)
Type of glaucoma, *n* (%)	
Normal	40 (47%)
Primary open angle glaucoma	11 (13%)
Primary open angle glaucoma suspect	9 (10%)
Acute primary angle closure glaucoma	2 (2%)
Primary angle closure glaucoma	8 (9%)
Primary angle closure	7 (8%)
Primary angle closure suspect	9 (10%)
Average IOP, mm Hg (SD)	14.8 (3.2)
Number of IOP-lowering medications, *n* (%)	
0	69 (80%)
1	10 (12%)
2	4 (5%)
3	3 (3%)
Previous ocular surgery, *n* (%)	
Argon laser trabeculoplasty	1 (1%)
Cataract extraction	3 (3%)
Laser peripheral iridotomy	6 (7%)
Laser-assisted keratomileusis	4 (5%)
Conjunctival abnormality, *n* (%)	7 (8%)
Corneal abnormality, *n* (%)	
Punctate epithelial erosions	5 (6%)
Punctate epithelial keratopathy	3 (3%)
Superficial punctate keratitis	31 (36%)
Others	7 (8%)
Lens abnormality, *n* (%)	
Cataract	53 (62%)
Posterior chamber intraocular lens	3 (3%)
Spaeth grading by the 1st gonioscopic examiner, *n* (%)	
A: anterior to Schwalbe's line	12 (14%)
B: between Schwalbe's line and scleral spur	12 (14%)
C: scleral spur visible	19 (22%)
D: ciliary body	26 (30%)
E: beyond 0.1 mm of ciliary body	17 (20%)
Presence of PAS^a^ (%)	11 (13%)

SD: standard deviation; IOP: intraocular pressure; PAS: peripheral anterior synechiae.

^a^Missing 2 data points.

**Table 2:** Gonioscopy “pooled” interobserver agreement for angle classification (kappa [95% confidence interval]).

Kappa [95% CI]	*N*	Agree			Disagree *n* (%)
		Overall *n* (%)	Narrow *n*	Open *n*	
*Visit 1 *					
0.66 [0.50, 0.83]	86	73 (85%)	22	51	13 (15%)

*Visit 2 *					
0.69 [0.50, 0.89]	71	63 (89%)	13	50	8 (11%)

CI: confidence interval.

The kappa criteria were <0.2 poor; 0.21 to 0.40 fair; 0.41 to 0.60 moderate; 0.61 to 0.80 good; and >0.80 excellent.

**Table 3:** Gonioscopic intraobserver (intervisit) agreement for angle classification by examiner (kappa [95% confidence interval]).

Examiner	Kappa [95% CI]	*N*	Agree			Disagree *n* (%)
			Overall *n* (%)	Narrow *n*	Open *n*	
V	0.67 [0.10, 1.00]	6	5 (83%)	3	2	1 (17%)
W	0.72 [0.43, 1.00]	27	24 (89%)	6	18	3 (11%)
X	0.86 [0.68, 1.00]	32	30 (94%)	10	20	2 (6%)
Y	0.68 [0.43, 0.94]	36	31 (86%)	9	22	5 (14%)
Z	0.53 [0.07, 1.00]	41	38 (93%)	2	36	3 (7%)
*Combined*	*0.74* [*0.62, 0.87*]	*142*	*128 (90%)*	*30*	*98*	*14 (10%)*

CI: confidence interval.

The kappa criteria were <0.2 poor; 0.21 to 0.40 fair; 0.41 to 0.60 moderate; 0.61 to 0.80 good; and >0.80 excellent.

**Table 4:** Fourier domain anterior segment optical coherence tomography agreement for angle classification (kappa [95% confidence interval]).

Kappa [95% CI]	Agree				Disagree *n* (%)
	*N*	Overall *n* (%)	Narrow *n*	Open *n*	
*Interobserver, visit 1*					
0.73 [0.59, 0.88]	85	74 (87%)	30	44	11 (13%)

*Interobserver, visit 2*					
0.58 [0.39, 0.77]	72	58 (81%)	19	39	14 (19%)

*Intraobserver (intervisit), reader 1*					
0.57 [0.37, 0.77]	71	57 (80%)	18	39	14 (20%)

*Intraobserver (intervisit), reader 2*					
0.83 [0.69, 0.96]	71	65 (92%)	26	39	6 (8%)

CI: confidence interval.

Kappa criteria: <0.2 poor; 0.21 to 0.40 fair; 0.41 to 0.60 moderate; 0.61 to 0.80 good; and >0.80 excellent.

**Table 5:** Agreement (%) between pairs of gonioscopic examiners and Fourier domain anterior segment optical coherence tomography readers at each visit.

Examiner-reader pair	Kappa[95% CI]	Agree			Disagree	
		Overall *n* (%)	Narrow *n*	Open *n*	G: narrow A: open *n* (%)	G: open A: narrow *n* (%)
	*Visit 1* (*N* = 85)					
1st examiner versus reader 1	0.48 [0.28, 0.67]	65 (76%)	47	18	5 (6%)	15 (18%)
1st examiner versus reader 2	0.43 [0.25, 0.61]	62 (73%)	43	19	4 (5%)	19 (22%)
2nd examiner versus reader 1	0.63 [0.46, 0.80]	70 (82%)	45	25	7 (8%)	8 (9%)
2nd examiner versus reader 2	0.57 [0.39, 0.74]	67 (79%)	41	26	6 (7%)	12 (14%)

	*Visit 2* (*N* = 71)					
1st examiner versus reader 1	0.46 [0.23, 0.69]	56 (78%)	45	11	4 (6%)	11 (15%)
1st examiner versus reader 2	0.45 [0.25, 0.66]	54 (71%)	41	13	2 (3%)	15 (21%)
2nd examiner versus reader 1	0.42 [0.19, 0.65]	54 (71%)	42	12	7 (10%)	10 (14%)
2nd examiner versus reader 2	0.53 [0.33, 0.73]	56 (78%)	40	16	3 (4%)	12 (17%)

CI: confidence interval.

G: gonioscopic examination; A: anterior segment optical coherence tomography.

Kappa criteria: <0.2 poor; 0.21 to 0.40 fair; 0.41 to 0.60 moderate; 0.61 to 0.80 good; and >0.80 excellent.

## References

[1] Mansouri K., Burgener N. D., Bagnoud M., Shaarawy T. (2009). A prospective ultrasound biomicroscopy evaluation of changes in anterior segment morphology following laser iridotomy in European eyes. *Eye*.

[2] Sakata L. M., Lavanya R., Friedman D. S. (2008). Assessment of the scleral spur in anterior segment optical coherence tomography images. *Archives of Ophthalmology*.

[3] Blieden L. S., Chuang A. Z., Baker L. A. (2015). Optimal number of angle images for calculating anterior angle volume and iris volume measurements. *Investigative Ophthalmology and Visual Science*.

[4] Cumba R. J., Radhakrishnan S., Bell N. P. (2012). Reproducibility of scleral spur identification and angle measurements using fourier domain anterior segment optical coherence tomography. *Journal of Ophthalmology*.

[5] Rigi M., Blieden L. S., Nguyen D. (2014). Trabecular-iris circumference volume in open angle eyes using swept-source fourier domain anterior segment optical coherence tomography. *Journal of Ophthalmology*.

[6] Hu C. X., Mantravadi A., Zangalli C. (2016). Comparing gonioscopy with visante and cirrus optical coherence tomography for anterior chamber angle assessment in glaucoma patients. *Journal of Glaucoma*.

[7] Sakata L. M., Lavanya R., Friedman D. S. (2008). Comparison of gonioscopy and anterior segment ocular coherence tomography in detecting angle closure in different quadrants of the anterior chamber angle. *Ophthalmology*.

[8] Tay E. L. T., Yong V. K. Y., Lim B. A., Sia S., Wong E. P. Y., Yip L. W. L. (2015). Agreement of angle closure assessments between gonioscopy, anterior segment optical coherence tomography and spectral domain optical coherence tomography. *International Journal of Ophthalmology*.

[9] Baskaran M., Aung T., Friedman D. S., Tun T. A., Perera S. A. (2012). Comparison of EyeCam and anterior segment optical coherence tomography in detecting angle closure. *Acta Ophthalmologica*.

[10] Campbell P., Redmond T., Agarwal R., Marshall L. R., Evans B. J. W. (2015). Repeatability and comparison of clinical techniques for anterior chamber angle assessment. *Ophthalmic and Physiological Optics*.

[11] Park S. B., Sung K. R., Kang S. Y., Jo J. W., Lee K. S., Kook M. S. (2011). Assessment of narrow angles by gonioscopy, Van Herick method and anterior segment optical coherence tomography. *Japanese Journal of Ophthalmology*.

[12] Quek D. T., Narayanaswamy A. K., Tun T. A. (2012). Comparison of two spectral domain optical coherence tomography devices for angle-closure assessment. *Investigative Ophthalmology & Visual Science*.

[13] Kansara S., Blieden L. S., Chuang A. Z. (2016). Effect of laser peripheral iridotomy on anterior chamber angle anatomy in primary angle closure spectrum eyes. *Journal of Glaucoma*.

[14] Spaeth G. L. (1971). The normal development of the human anterior chamber angle: a new system of descriptive grading. *Transactions of the Ophthalmological Societies of the United Kingdom*.

[15] Spaeth G. L., Aruajo S., Azuara A., Ritch R., Drews R., Gaasterland D. (1995). Comparison of the configuration of the human anterior chamber angle, as determined by the Spaeth gonioscopic grading system and ultrasound biomicroscopy. *Transactions of the American Ophthalmological Society*.

[16] Gold M. E., Kansara S., Nagi K. S. (2013). Age-related changes in trabecular meshwork imaging. *BioMed Research International*.

[17] Usui T., Tomidokoro A., Mishima K. (2011). Identification of Schlemm's canal and its surrounding tissues by anterior segment Fourier domain optical coherence tomography. *Investigative Ophthalmology & Visual Science*.

[18] Altman D. G. (1991). *Practical Statistics for Medical Research*.

[19] Radhakrishnan S., Goldsmith J., Huang D. (2005). Comparison of optical coherence tomography and ultrasound biomicroscopy for detection of narrow anterior chamber angles. *Archives of Ophthalmology*.

[20] Barkana Y., Dorairaj S. K., Gerber Y., Liebmann J. M., Ritch R. (2007). Agreement between gonioscopy and ultrasound biomicroscopy in detecting iridotrabecular apposition. *Archives of Ophthalmology*.

[21] Nolan W. P., See J. L., Chew P. T. K. (2007). Detection of primary angle closure using anterior segment optical coherence tomography in Asian eyes. *Ophthalmology*.

